# Supplementary material for: Cardiomyocyte BRAF is a key signalling intermediate in cardiac hypertrophy in mice
Source: Clin Sci (Lond). 2022 Nov 21;136(22):1661–81. doi: 10.1042/CS20220607 (PMC9679367; doi:10.1042/CS20220607)
Supplement: Supplementary Figure S1 and Tables S1-S7 [file CS-2022-0607_supp.pdf]

SUPPLEMENTARY DATA

Cardiomyocyte BRAF is a key signalling intermediate in cardiac hypertrophy in mice.

Hajed O. Alharbi[1], Michelle A. Hardyman[1], Joshua J. Cull[1], Thomais Markou[1], Susanna T.E. Cooper[2], Peter E. Glennon[3], Stephen J. Fuller[1], Peter H. Sugden[1] and Angela Clerk[1]

[1] School of Biological Sciences, University of Reading, Reading, UK

[2] Molecular and Clinical Sciences Institute, St. George's University of London, London, UK.

[3] University Hospitals Coventry and Warwickshire, University Hospital Cardiology Department, Clifford Bridge Road, Coventry, UK.

Supplementary Figure S1. Heart weight to body weight ratios.

Supplementary Table S1. Mouse weights.

Supplementary Table S2. Primers for genotyping and confirmation of recombination.

Supplementary Table S3. qPCR primers.

Supplementary Table S4. Echocardiography data for male hemizygous Cre mice treated without/with angiotensin II.

Supplementary Table S5. Echocardiography data for male BRAFKO mice treated without/with angiotensin II.

Supplementary Table S6. Echocardiography data for male BRAFKO mice treated without/with phenylephrine.

Supplementary Table S7. Echocardiography data for female BRAFKO mice treated without/with phenylephrine.

Supplementary Figure S1. Ratios of heart weight (HW) to body weight (BW). Male (**A**) or female (**B**) BRAF$^{fl/fl}$/Cre$^{+/-}$ mice were treated with corn-oil (CO) or tamoxifen in corn-oil (Tx) 4 days before minipumps were implanted for delivery of PBS or acidified PBS (Vehicle) or 0.8 mg/kg/d angiotensin II in AcPBS (AngII), or 40 mg/kg/d phenylephrine (PE) for 7 d. Heart and body weights were taken at the end of the experiment.

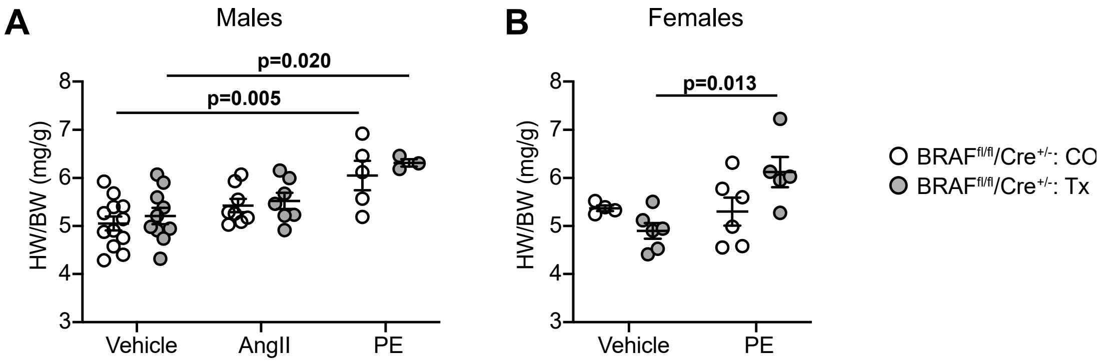

Supplementary Table S1. Mouse weights (g). Mice were allocated to groups on a random basis and were treated with corn-oil (CO) or tamoxifen in corn-oil (Tx) with acidified PBS (AcPBS) or 0.8 mg/kg/d angiotensin II (AngII), or with PBS or 40 mg/kg/d phenylephrine in PBS (PE). Weights were taken at the start of the study with the first baseline echocardiogram (Start), immediately after minipump surgery, and when mice were culled (End). Weights post-surgery and at the end included the minipumps. Male mice were 7-8 weeks at the start of the experiment; female mice were 9-10 weeks at the start of the experiment.

Study	Condition	Start		Post-minipump		End		
		**Mean**	**SEM**	**Mean**	**SEM**	**Mean**	**SEM**	**n**
**Cre$^{+/-}$**								
AngII (males)	CO/AcPBS	22.45	1.07	24.60	0.55	25.28	0.68	4
(echocardiography	Tx/AcPBS	22.14	1.18	23.80	1.36	24.08	1.37	5
+ biochemistry)	CO/AngII	22.28	1.21	23.35	0.95	23.95	0.79	6
	Tx/AngII	22.82	1.07	23.92	0.82	24.08	0.77	5
**BRAF$^{fl/fl}$/Cre$^{+/-}$**								
AngII (males)	CO/AcPBS	24.01	0.56	25.01	0.48	25.10	0.44	10
(echocardiography	Tx/AcPBS	24.70	0.48	25.93	0.44	26.13	0.40	8
+ biochemistry)	CO/AngII	25.17	0.38	26.60	0.33	26.12	0.64	10
	Tx/AngII	23.83	0.54	25.10	0.45	24.93	0.37	10
AngII (males)	CO/AcPBS	23.65	0.54	26.36	0.49	26.96	0.57	8
(histology)	Tx/AcPBS	22.33	0.31	25.23	0.30	26.05	0.17	4
	CO/AngII	24.29	0.28	26.60	0.26	26.80	0.37	7
	Tx/AngII	22.88	0.70	26.30	0.56	26.36	0.58	5
PE (males)	CO/PBS	23.77	0.55	25.07	0.36	25.54	0.39	7
	Tx/PBS	23.55	0.41	25.49	0.38	25.71	0.43	8
	CO/PE	23.89	0.75	25.13	0.63	24.73	0.61	8
	Tx/PE	23.06	0.75	24.29	0.53	24.51	0.44	8
PE (females)	CO/PBS	19.64	0.52	21.10	0.58	21.31	0.58	9
	Tx/PBS	19.19	0.39	20.96	0.28	20.99	0.21	10
	CO/PE	19.19	0.58	20.20	0.49	20.47	0.43	10
	Tx/PE	19.64	0.60	20.77	0.40	20.74	0.35	10

Supplementary Table S2. Primers for genotyping and confirmation of recombination.

Mouse strain	DNA	Forward primer	Reverse primer	Annealing temp.
**Genotyping**				
BRAF$^{fl/fl}$	gDNA	GCATAGCGCATATGCTCACA	CCATGCTCTAACTAGTGCTG	57°C
Cre^{-}	gDNA	TCTATTGCACACAGCAATCCA	CCAACTCTTGTGAGAGGAGCA	52°C
Cre^{+}	gDNA	TCTATTGCACACAGCAATCCA	CCAGCATTGTGAGAACAAGG	52°C
**Recombination**				
BRAF$^{fl/fl}$ (exons 9-13)	cDNA	TTGATTTTGAGCCTGGCCCAGTG	GTGCAGTCTGCCGAGCAATATC	57°C
BRAF$^{fl/fl}$ (exons 10-13)	cDNA	GTCATCTTCTTCCTCATCCTCG	GTGCAGTCTGCCGAGCAATATC	57°C

Supplementary S3. qPCR primers.

Gene Symbol	Accession No.	Sense Primer (5'→3')	Antisense Primer (5'→3')
Col1a1	NM_007742	TCGTGGCTTCTCTGGTCTC	CCGTTGAGTCCGTCTTTGC
Col4a1	NM_009931.2	TGTGGGCCAGCCAGGCATTG	CAGGGGGTCCGATCGCTCCA
Ctgf	NM_010217	GCACACCGCACAGAACCA	ATGGCAGGCACAGGTCTTG
Ddr2	NM_022563.2	GCACTTGGTGAATTAATTAGAATCCTG	GGACAACTAAATGGTCCCTCCC
Edn1	NM_007913	GCCTTCGCTCACTCCACTA	GCTGGGATTGGTAGGTGGTA
Fn1	NM_010233	AAGAGGACGTTGCAGAGCTA	AGACACTGGAGACACTGACTAA
Gapdh	NM_008084.2	TCACCACCATGGAGAAGGC	GCTAAGCAGTTGGTGGTGCA
Hif1a	NM_010431	GATGTAATGTTTCCCTCTTCTAATGA	GCAGGATCAGCACTACTTCG
Il6	NM_031168	TCCATCCAGTTGCCTTCTTG	GGTCTGTTGGGAGTGGTATC
Il1b	NM_008361	CAACCAACAAGTGATATTCTCCAT	GGGTGTGCCGTCTTTCATTA
Lox	NM_001286181	GACATTCGCTACACAGGACAT	AACACCAGGTACGGCTTTATC
Myh6	NM_010856	CGAGCTGGATGAGGCGGAG	TCTGCTGGAGAGGTTATTCCTCG
Myh7	NM_080728	CATGCCAACCGTATGGCTG	GTTCCACGATGGCGATGTTC
Nppa	NM_008725	GATGGATTTCAAGAACCTGCTAGA	CTTCCTCAGTCTGCTCACTCA
Nppb	NM_008726	TCCAGCAGAGACCTCAAAATTC	CAGTGCGTTACAGCCCAAA
Postn	NM_015784	TTCCTCTCCTGCCCTTATATGC	CCTGATCCCGACCCCTGAT
NOX1	NM_172203	CATCCCTTCACTCTGACTTCTG	GCAG[illegible]GTCCTTATG
NOX2 (Cybb)	NM_007807	GCTATGAGGTGGTGATGTTAGT	GTTTCAGACTGGTGGCATTATC
NOX4	NM_015760	GGAAGCCCATTTGAGGAGTC	TCCAGTCATCCAGTAGAGTGTT
Tgfb1	NM_011577	TGGACACACAGTACAGCAAG	GTAGTAGACGATGGGCAGTG

Supplementary Table S4. Echocardiography data for hemizygous Cre$^{+/-}$ male mice treated without/with angiotensin II. Male Cre$^{+/-}$ mice (7-8 weeks) were treated with corn-oil (CO) or tamoxifen in corn-oil (Tx) 4 days before minipumps were implanted for delivery of acidified PBS (AcPBS) or 0.8 mg/kg/d angiotensin II in AcPBS (AngII) for 7 d. Echocardiograms were taken prior to tamoxifen treatment (Baseline) and 7 d after minipump implantation. Echocardiograms were analysed using VevoLab software. LV, left ventricle; ID, internal diameter; AW, anterior wall; PW, posterior wall; WT, wall thickness (AW+PW); EDV, end diastolic volume; ESV, end systolic volume; EDLVM, end diastolic LV mass; ESLVM, end systolic LV mass.

* Data collected from B-mode images of long axis views. Other parameters were measured from M-mode images of short axis views.

**7 d**	**CO/AcPBS (n=4)**		**Tx/AcPBS (n=5)**		**CO/AngII (n=6)**		**Tx/AngII (n=5)**	
	Mean	SEM	Mean	SEM	Mean	SEM	Mean	SEM
Heart Rate (bpm)	470	24	446	9	471	21	479	17
LVID;s (mm)	3.380	0.113	3.254	0.125	2.962	0.213	2.746	0.114
LVID;d (mm)	4.356	0.131	4.215	0.192	3.873	0.150	3.757	0.177
LVAW;s (mm)	0.887	0.050	0.958	0.023	0.993	0.040	1.091	0.041
LVAW;d (mm)	0.736	0.029	0.747	0.006	0.785	0.022	0.866	0.027
LVPW;s (mm)	0.931	0.059	0.939	0.064	1.096	0.064	1.096	0.041
LVPW;d (mm)	0.698	0.039	0.722	0.032	0.855	0.069	0.829	0.077
EDV (µl)*	59.40	4.98	59.46	8.36	46.83	4.16	46.53	5.32
ESV (µl)*	32.26	2.96	29.47	2.90	24.92	2.16	21.44	2.17
EDLVM (mg)*	59.81	1.42	57.65	2.40	70.02	1.88	66.48	1.15
ESLVM (mg)*	62.78	1.19	61.35	3.75	71.50	2.01	70.50	0.86
Stroke Volume (µl)*	27.14	2.29	29.99	6.04	21.91	2.62	25.09	3.15
Ejection Fraction (%)*	45.65	1.41	49.09	3.20	46.35	2.54	53.26	0.68
Fractional Shortening (%)*	22.16	2.20	24.68	2.32	23.94	1.15	26.02	3.11
Cardiac Output (ml/min)*	12.37	0.88	12.85	2.73	9.78	1.17	10.92	1.07
GLS (%)*	-16.60	2.17	-15.87	0.62	-14.88	1.61	-17.53	1.28
GCS (%)*	-17.30	1.23	-18.98	1.48	-18.87	1.14	-20.93	1.36

Supplementary Table S5. Echocardiography data for male mice with cardiomyocyte BRAF knockout treated without/with angiotensin II. Male $BRAF^{fl/fl}/Cre^{+/-}$ mice (7-8 weeks) were treated with corn-oil (CO) or tamoxifen in corn-oil (Tx) 4 days before minipumps were implanted for delivery of acidified PBS (AcPBS) or 0.8 mg/kg/d angiotensin II in AcPBS (AngII) for 7 d. Echocardiograms were taken prior to tamoxifen treatment (Baseline) and 7 d after minipump implantation. Echocardiograms were analysed using VevoLab software. LV, left ventricle; ID, internal diameter; AW, anterior wall; PW, posterior wall; WT, wall thickness (AW+PW); EDV, end diastolic volume; ESV, end systolic volume; EDLVM, end diastolic LV mass; ESLVM, end systolic LV mass.

* Data collected from B-mode images of long axis views. Other parameters were measured from M-mode images of short axis views.

**Baseline**	**CO/AcPBS (n=10)**		**Tx/AcPBS (n=8)**		**CO/AngII (n=10)**		**Tx/AngII (n=10)**	
	Mean	SEM	Mean	SEM	Mean	SEM	Mean	SEM
Heart Rate (bpm)	463	17	476	10	463	10	462	10
LVID;s (mm)	2.929	0.163	3.020	0.093	3.016	0.128	3.165	0.043
LVID;d (mm)	3.984	0.110	3.978	0.076	3.985	0.097	4.157	0.031
LVAW;s (mm)	0.981	0.024	0.943	0.018	0.935	0.016	0.949	0.013
LVAW;d (mm)	0.728	0.015	0.717	0.011	0.726	0.015	0.738	0.012
LVPW;s (mm)	0.967	0.036	0.909	0.032	0.939	0.016	0.941	0.028
LVPW;d (mm)	0.685	0.020	0.670	0.019	0.691	0.014	0.670	0.018
EDV (µl)*	51.36	2.91	52.97	1.85	49.45	2.35	47.74	1.56
ESV (µl)*	26.95	2.24	28.21	1.53	27.99	2.20	25.19	1.30
EDLVM (mg)*	55.87	2.19	53.42	1.77	56.67	2.28	52.32	1.96
ESLVM (mg)*	58.39	2.02	56.68	1.75	58.87	2.11	55.19	1.96
Stroke Volume (µl)	35.46	1.56	33.31	1.19	33.52	0.96	36.74	1.05
Ejection Fraction (%)	52.30	3.72	48.47	1.88	49.01	2.51	47.92	1.31
Fractional Shortening (%)	27.04	2.54	24.21	1.12	24.61	1.56	23.88	0.79
Cardiac Output (ml/min)	16.26	0.64	15.85	0.68	15.46	0.51	16.95	0.57
**7 d**	**CO/AcPBS (n=10)**		**Tx/AcPBS (n=8)**		**CO/AngII (n=10)**		**Tx/AngII (n=10)**	
	Mean	SEM	Mean	SEM	Mean	SEM	Mean	SEM
Heart Rate (bpm)	488	20	500	9	512	15	489	21
LVID;s (mm)	3.000	0.118	2.986	0.128	2.515	0.147	2.651	0.189
LVID;d (mm)	3.998	0.087	4.024	0.104	3.581	0.125	3.771	0.133
LVAW;s (mm)	0.976	0.026	0.980	0.034	1.160	0.043	1.122	0.081
LVAW;d (mm)	0.751	0.020	0.715	0.015	0.907	0.030	0.867	0.043
LVPW;s (mm)	0.963	0.043	0.942	0.019	1.217	0.049	1.157	0.060
LVPW;d (mm)	0.699	0.031	0.662	0.018	0.899	0.032	0.815	0.049
EDV (µl)*	47.14	2.80	51.97	3.36	39.36	2.50	37.87	3.43
ESV (µl)*	28.22	2.43	27.17	2.03	20.96	1.74	19.66	2.72
EDLVM (mg)*	52.97	1.41	55.25	1.72	69.40	2.53	64.24	4.73
ESLVM (mg)*	55.55	1.77	59.09	1.71	72.32	2.60	67.32	5.07
Stroke Volume (µl)	34.43	2.01	36.09	1.85	30.63	1.78	33.95	1.90
Ejection Fraction (%)	49.69	3.07	51.21	2.67	57.88	3.07	57.26	4.29
Fractional Shortening (%)	25.18	1.93	26.00	1.69	30.46	2.28	30.42	3.09

Supplementary Table S6. Echocardiography data for male mice with cardiomyocyte BRAF knockout treated without/with phenylephrine. Male $BRAF^{fl/fl}/Cre^{+/-}$ mice (7-8 weeks) were treated with corn-oil (CO) or tamoxifen in corn-oil (Tx) 4 days before minipumps were implanted for delivery of PBS or 40 mg/kg/d phenylephrine in PBS (PE) for 7 d. Echocardiograms were taken prior to tamoxifen treatment (Baseline) and 7 d after minipump implantation. Echocardiograms were analysed. LV, left ventricle; ID, internal diameter; AW, anterior wall; PW, posterior wall; WT, wall thickness (AW+PW); EDV, end diastolic volume; ESV, end systolic volume; EDLVM, end diastolic LV mass; ESLVM, end systolic LV mass; GLS, global longitudinal strain; GCS, global circumferential strain.

* Data collected from B-mode images were analysed using VevoStrain software. Other parameters were measured from M-mode images of short axis views using VevoLab software.

**Baseline**	**CO/PBS (n=7)**		**Tx/PBS (n=8)**		**CO/PE (n=8)**		**Tx/PE (n=8)**	
	Mean	SEM	Mean	SEM	Mean	SEM	Mean	SEM
Heart Rate (bpm)	457	10	469	12	459	12	474	15
LVID;s (mm)	2.697	0.125	2.421	0.113	2.723	0.096	2.315	0.127
LVID;d (mm)	3.908	0.126	3.729	0.102	3.920	0.091	3.653	0.088
LVAW;s (mm)	1.014	0.016	1.025	0.018	1.012	0.024	1.054	0.030
LVAW;d (mm)	0.744	0.012	0.760	0.007	0.752	0.012	0.773	0.012
LVPW;s (mm)	1.003	0.018	1.093	0.037	1.011	0.030	1.058	0.037
LVPW;d (mm)	0.686	0.024	0.711	0.015	0.701	0.019	0.704	0.020
EDV (µl)*	46.42	4.29	42.65	2.43	46.10	1.52	40.69	3.20
ESV (µl)*	19.01	1.75	16.72	1.88	20.49	1.51	15.52	2.62
EDLVM (mg)*	50.25	2.32	49.31	1.12	49.51	1.80	49.24	1.99
ESLVM (mg)*	51.80	2.52	50.72	1.27	50.67	1.79	50.09	2.30
Stroke Volume (µl)*	27.41	2.77	25.94	1.31	25.61	1.40	25.17	1.47
Ejection Fraction (%)*	59.43	1.75	61.25	3.04	55.27	2.64	63.52	4.28
Fractional Shortening (%)*	31.23	1.70	34.86	2.59	28.28	2.97	36.95	3.67
Cardiac Output (ml/min)*	12.30	1.26	12.13	0.70	11.46	0.83	11.67	0.57
GLS (%)*	-20.62	1.02	-21.27	1.02	-19.03	0.88	-20.52	1.72
GCS (%)*	-21.43	1.69	-24.66	1.48	-21.89	1.24	-25.33	1.66
**7 d**	**CO/PBS (n=7)**		**Tx/PBS (n=8)**		**CO/PE (n=8)**		**Tx/PE (n=8)**	
	Mean	SEM	Mean	SEM	Mean	SEM	Mean	SEM
Heart Rate (bpm)	485	18	513	9	470	15	465	17
LVID;s (mm)	2.622	0.130	2.636	0.118	2.137	0.151	1.920	0.126
LVID;d (mm)	3.841	0.119	3.917	0.105	3.505	0.121	3.307	0.077
LVAW;s (mm)	1.041	0.028	1.101	0.031	1.276	0.064	1.297	0.076
LVAW;d (mm)	0.771	0.012	0.801	0.017	0.865	0.038	0.917	0.054
LVPW;s (mm)	1.029	0.028	1.072	0.032	1.301	0.095	1.405	0.099
LVPW;d (mm)	0.697	0.027	0.708	0.024	0.974	0.089	1.000	0.094
EDV (µl)*	47.61	3.65	48.06	3.10	34.69	3.07	32.84	2.94
ESV (µl)*	18.74	1.97	19.72	2.10	10.64	1.45	9.91	1.47
EDLVM (mg)*	53.23	2.60	51.93	1.41	68.34	3.36	67.43	4.89
ESLVM (mg)*	55.29	2.98	53.82	1.50	70.73	3.65	69.15	5.33
Stroke Volume (µl)*	28.87	2.58	28.33	1.50	24.04	1.83	22.93	1.83
Ejection Fraction (%)*	60.44	2.76	59.13	2.42	70.04	1.95	70.30	2.48
Fractional Shortening (%)*	32.02	3.56	32.85	2.40	40.67	2.84	41.67	1.90
Cardiac Output (ml/min)*	13.67	0.87	13.83	0.92	11.29	0.92	10.65	1.00
GLS (%)*	-19.70	1.55	-20.90	1.18	-23.33	1.05	-19.44	1.44
GCS (%)*	-22.79	1.69	-23.22	0.75	-28.33	1.33	-28.57	2.05

Supplementary Table S7. Echocardiography data for female mice with cardiomyocyte BRAF knockout treated without/with phenylephrine. Female $BRAF^{fl/fl}/Cre^{+/-}$ mice (9-10 weeks) were treated with corn-oil (CO) or tamoxifen in corn-oil (Tx) 4 days before minipumps were implanted for delivery of PBS or 40 mg/kg/d phenylephrine in PBS (PE) for 7 d. Echocardiograms were taken prior to tamoxifen treatment (Baseline) and 7 d after minipump implantation. Echocardiograms were analysed. LV, left ventricle; ID, internal diameter; AW, anterior wall; PW, posterior wall; WT, wall thickness (AW+PW); EDV, end diastolic volume; ESV, end systolic volume; EDLVM, end diastolic LV mass; ESLVM, end systolic LV mass; GLS, global longitudinal strain; GCS, global circumferential strain.
* Data collected from B-mode images were analysed using VevoStrain software. Other parameters were measured from M-mode images of short axis views using VevoLab software.

**Baseline**	**CO/PBS (n=9)**		**Tx/PBS (n=10)**		**CO/PE (n=10)**		**Tx/PE (n=10)**	
	Mean	SEM	Mean	SEM	Mean	SEM	Mean	SEM
Heart Rate (bpm)	457	17	464	7	465	14	452	10
LVID;s (mm)	2.576	0.104	2.556	0.061	2.569	0.113	2.636	0.089
LVID;d (mm)	3.726	0.067	3.632	0.055	3.671	0.113	3.743	0.069
LVAW;s (mm)	0.941	0.008	0.948	0.011	0.959	0.021	0.917	0.014
LVAW;d (mm)	0.713	0.008	0.716	0.008	0.696	0.015	0.700	0.009
LVPW;s (mm)	0.938	0.026	0.917	0.012	0.907	0.018	0.933	0.018
LVPW;d (mm)	0.639	0.011	0.657	0.018	0.650	0.021	0.650	0.011
EDV (μl)*	38.34	2.64	40.18	1.15	39.53	2.96	41.20	2.10
ESV (μl)*	15.70	2.42	17.31	1.24	17.42	2.07	18.52	1.57
EDLVM (mg)*	43.46	1.48	43.45	1.21	43.65	1.99	43.69	1.57
ESLVM (mg)*	44.50	1.53	44.70	1.61	45.34	2.14	44.76	1.66
Stroke Volume (μl)*	22.64	0.99	22.87	1.01	22.11	1.08	22.68	0.72
Ejection Fraction (%)*	60.72	3.79	57.11	2.54	56.94	2.25	55.50	1.68
Fractional Shortening (%)*	33.71	2.86	31.65	2.09	31.15	2.09	30.91	1.62
Cardiac Output (ml/min)*	10.34	0.50	10.49	0.56	10.11	0.62	9.93	0.34
GLS (%)*	-21.15	1.69	-19.50	1.33	-18.41	0.78	-18.69	0.76
GCS (%)*	-21.76	1.54	-22.21	1.36	-22.76	1.23	-20.35	0.96
**7 d**	**CO/PBS (n=9)**		**Tx/PBS (n=10)**		**CO/PE (n=10)**		**Tx/PE (n=10)**	
	Mean	SEM	Mean	SEM	Mean	SEM	Mean	SEM
Heart Rate (bpm)	487	16	478	13	471	13	447	11
LVID;s (mm)	2.570	0.142	2.508	0.093	1.995	0.118	1.946	0.109
LVID;d (mm)	3.786	0.106	3.632	0.045	3.310	0.090	3.293	0.098
LVAW;s (mm)	1.004	0.025	1.001	0.017	1.183	0.058	1.261	0.086
LVAW;d (mm)	0.739	0.021	0.726	0.010	0.821	0.031	0.900	0.052
LVPW;s (mm)	1.004	0.045	0.958	0.021	1.249	0.076	1.282	0.096
LVPW;d (mm)	0.674	0.014	0.677	0.022	0.883	0.076	0.919	0.090
EDV (μl)*	36.36	2.52	38.51	1.78	30.70	2.06	31.39	2.33
ESV (μl)*	15.89	2.34	15.81	1.40	11.57	1.00	9.42	0.73
EDLVM (mg)*	49.56	2.09	51.12	1.66	57.60	5.03	63.09	5.35
ESLVM (mg)*	51.19	2.05	52.90	1.90	59.42	5.29	64.74	5.32
Stroke Volume (μl)*	20.47	1.37	22.71	1.02	19.13	1.49	21.97	1.85
Ejection Fraction (%)*	57.50	4.36	59.04	2.27	61.68	2.44	69.25	1.90
Fractional Shortening (%)*	30.83	3.50	32.53	1.88	33.77	2.45	43.86	2.60
Cardiac Output (ml/min)*	9.91	0.85	11.01	0.67	8.64	0.69	9.65	1.03
GLS (%)*	-18.45	1.86	-19.59	0.72	-19.67	1.55	-20.51	1.55
GCS (%)*	-23.21	1.78	-21.77	1.53	-26.72	1.78	-28.36	1.77
